# Comparative treatments of a green tattoo ink with Ruby, Nd:YAG nano- and picosecond lasers in normal and array mode

**DOI:** 10.1038/s41598-022-07021-w

**Published:** 2022-03-04

**Authors:** Daniele Cecchetti, Elvira Maria Bauer, Ettore Guerriero, Simona Sennato, Pietro Tagliatesta, Marco Tagliaferri, Luca Cerri, Marilena Carbone

**Affiliations:** 1 Department of Chemical Science and Technologies, University of Rome Tor Vergata, Via della Ricerca Scientifica, 1, 00133 Rome, Italy; 2 Institute of Structure of Matter, Italian National Research Council (CNR-ISM), Via Salaria km 29.3, 00015 Monterotondo, RM Italy; 3 Institute of Atmospheric Pollution Research, Italian National Research Council (CNR-IIA), Via Salaria km 29.3, 00015 Monterotondo, RM Italy; 4 Institute of Complex Systems, Italian National Research Council (CNR‑ISC) Sapienza Unit, and Physics Department, Sapienza University, P.le A. Moro 5, 00185 Rome, Italy; 5 El.En. S.P.A., Via Baldanzese 17, 50041 Calenzano, FI Italy; 6 Quanta System S.P.A., Via Acquedotto 109, 21017 Samarate, VA Italy

**Keywords:** Biomaterials, Soft materials, Bioanalytical chemistry, Organometallic chemistry, Bioinorganic chemistry, Solid-state chemistry, Risk factors, Health policy, Photobiology

## Abstract

The tattoos removal has become an issue upon spread of the tattooing practice worldwide and hindsight regrets. Lasers are typically used for the purpose, though some colours such as green are considered “recalcitrant” to the treatment. In the current investigation, we aim at determining the efficacy of removal of a green ink water dispersion, using 5 laser treatments: Nd:YAG nano- and picosecond lasers in normal and array mode and Ruby nanosecond laser, keeping the total irradiated energy constant. The UV–Vis spectroscopy of the treated samples indicate that Nd:YAG picosecond laser is most effective, and the Ruby nanosecond laser is the least efficient. Fragment compounds generated from the pigment and siloxanes are common to all treatments, whereas hydrocarbon emerge by a larger amount upon Nd:YAG nanosecond treatment. Fibres are formed upon picosecond treatments and when operating in array mode, and lamellae are achieved by Ruby nanosecond laser treatment. Residual particles suspensions are very heterogeneous upon nanosecond treatments.

## Introduction

Laser treatments are practical remedies in case of hindsight afterthoughts of unwanted (or no longer wanted) tattoos^1^. Their effectiveness is judged by comparing the discoloration of the tattooed skin before and after the treatments and the efficacy of tattoos’ removal is often colour dependent. Black tattoos have been reported to be successfully removed by several types of lasers such as Nd:YAG, Ruby or alexandrite lasers^2–4^, whereas, at the other extreme, green tattoos are the most recalcitrant ones^5,6^. When dealing with multicolour tattoos, a frequent outcome of the laser treatment is a variable bleaching which leaves residuals of different colours at various extents, with a consequent awkward final effect^7^. Recent reports indicate that picosecond Nd:YAG lasers are comparatively more effective than the nanosecond counterparts in the removal of green tattoos in Asians^8^. Moreover nano and picosecond Nd:YAG and Alexandrite lasers were used in order to remove multicoloured tattoo on Hartley guinea pigs^9^. In the latter case a Nd:YAG laser operated at 532 nm seems to be more effective in clearing red, yellow and orange coloured tattoos, regardless of the pulse duration, though safety is superior with picosecond lasers, and picosecond alexandrite is more effective on green and blue pigments. The conclusions, however, are shadowed by the composition of the green tattoo inks employed in this study, which are reportedly the same, i.e. they both contain Pigment Yellow 65 (C.I.11740, 2-[(4-methoxy-2-nitrophenyl)diazenyl]-N-(2-methoxyphenyl)-3-oxobutanamide), blue copper phthalocyanine (C.I.74160) and TiO_2_ (C.I.77891). Especially the latter may be responsible of misleading outcomes due to the “paradoxical darkening”^10^, thus hindering the actual results. Green and blue tattoo inks can be also based on one single pigment belonging to the family of copper phthalocyanine derivatives, i.e. PG36, (hexabromodecachloro copper phthalocyanine), PG7 (hexadecachloro copper phthalocyanine C.I. 74260) and PB15 (copper phthalocyanine, C.I.74160) though sometimes unproperly reported on the labels^11^. When treating unsubstituted copper phthalocyanine (PB15, C.I. 74160), Ruby laser is reported to be more efficient, because of a better absorption of the radiation by the chromophore^12^. However, the photochemical mechanism is probably a minority removal channel with respect to the photothermal and photomechanical (photoacoustic) ones^13^. Besides the efficacy in tattoo clearing, associated risks must be evaluated when choosing laser treatments, since recent studies outlined the production of toxic fragment molecules and potential harmful morphologies upon Ruby and Nd:YAG laser treatments^12,14^.

In the present study we offset efficacy against fragment production, morphology and associated potential risks in the treatment of a single pigment green ink, using 5 types of lasers setups. We used Nd:YAG picoseconds lasers and Nd:YAG and Ruby nanosecond lasers, which are the most common lasers for tattoo removal in dermatological practices. In addition, we operated the Nd:YAG lasers (both pico and nano) in normal mode, with spot size of 3 mm and in array mode or fractioned laser beam, i.e. by widening the laser beam to a spot size of 8 mm diameter and splitting it in an array of 180 nodes (Fig. SI1 of the Supplementary Information). The summary of the conditions adopted in the various treatments is reported in Table 1. In all cases we kept the total irradiated energy to 2 kJ, which was the amount of the energy necessary to discolour the Nd:YAGPico sample.

**Table 1:** Summary of the operational conditions of the 5 different laser treatments.

Sample	Laser	Wavelength (nm)	Pulse duration	Exposure time (min)	Spot size (mm)	Fluence	Array	Repet. rate (Hz)	Total energy (kJ)
RubyNano	Ruby	694	370 ns	42	3	8 J/cm^2^	No	1	2
Nd:YAGNano	Nd:YAG	532	3 ns	10	3	3 J/cm^2^	No	10	2
Nd:YAGNanoArray	Nd:YAG	532	3 ns	10	8*	0.15 mJ/n	180n	10	2
Nd:YAGPico	Nd:YAG	532	300 ps	12	3	2.5 J/cm^2^	No	10	2
Nd:YAGPicoArray	Nd:YAG	532	300 ps	12	8*	0.13 mJ/n	180n	10	2

*Outer size of the array. When using the array, the fluence is calculated as mJ/n where n = node.

The ink Green Concentrate (GC ink) by Eternal Ink was chosen for this investigation, since it reportedly contains a single green pigment, i.e. PG7 (though on the bottle label, it is mentioned PG36), thus avoiding overlapping and/or interfering effects of multiple pigments.

The laser treatments were applied to water dispersions of GC ink with a nominal concentration of 0.09 mg/ml. Photos of the vials before and after treatments are reported in Fig. SI2 of the Supplementary Information. Afterwards, we analyzed the treated samples by UV–Vis spectroscopy, for assessing the discoloration in terms of absorbance, by GC-mass spectrometry, SEM (scanning electron microscopy) and DLS (dynamic light scattering). The latter techniques aim at determining whether the same types of fragment molecules are produced upon different laser treatments as well as analyzing size and shape of the aggregates. Tattoo ink tends to localize in the papillary and reticular layers of the dermis^15^, both in fibroblasts, and in macrophages, the latter by capture-release and recapture mechanisms^16^, though at different extents. The morphology of the ink upon laser treatment may be of particular importance, because of possible size and shape interferences with the recapture-dependent mechanisms and contribute to the ink persistence. As for the array mode, it is reported that fractional Nd:YAG picosecond lasers on ex-vivo pigmented micropig skin accentuated laser-induced tissue reactions in wider areas of the epidermis and dermis, compared to single pulse treatment^17^. Therefore, we opted for probing this modality on the GC ink dispersions too.

## Results and discussion

### Nd:YAG picosecond lasers are most effective in discolouring the GC ink, Ruby nanosecond laser is the least efficient

**Figure 1:**
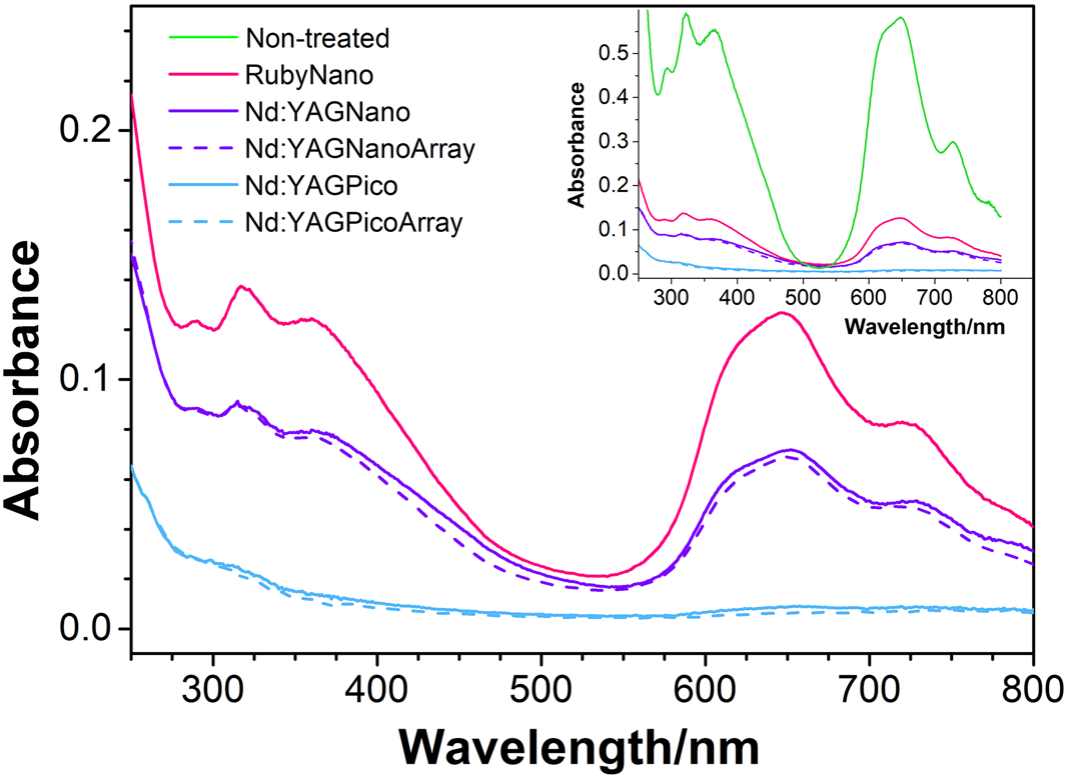
UV–Vis spectra of the GC ink dispersion upon laser treatments: red solid line = nanosecond ruby laser, violet solid line = nanosecond Nd:YAG, violet dashed line = nanosecond Nd:YAG with array, light blue solid line = picosecond Nd:YAG, light blue dashed line = picosecond Nd:YAG with array. In the inset, the same set of spectra is plotted along with the non-treated sample, reported with a green solid line.

The efficiency of the lasers in discoloration of the GC ink dispersions are evaluated by UV–Vis spectroscopy. In Fig. 1 the UV–Vis spectra of the GC ink dispersions after the laser treatments are reported. In addition, for comparison purposes, the same set is reported in the inset including the spectrum of the non-treated sample.

The spectrum of the GC ink before treatment displays the typical Soret and Q bands of copper phthalocyanines in the ranges 300–450 nm and 550–750 nm respectively. The spectral intensity depends on the aggregation state^18^ and increases when the size of aggregates allows formation of stable colloidal suspensions in the solvent^19^. Position and relative intensity of the absorption features at 293 nm, 322 nm, 366 nm, 620 nm, 651 nm, and 727 nm, indicates that the pigment present is PG7, the hexadecachloro copper phthalocyanine, instead of PG36, the hexabromo decachloro copper phthalocyanine indicated on the bottle label^14^. The laser treatments have effects of different extents on the absorption spectra. They determine an overall decrease of the absorption features in case of the picosecond treatments and a general decrease below 500 nm and above 550 nm in case of nanosecond treatments, with a marginal increase of 0.005 to 0.01 abs. units at 520 nm (i.e. 0.8% to 1.6% as compared to the peak intensity at 651 nm). The Nd:YAG picosecond laser is the most effective, causing an almost complete disappearance of the absorbances, i.e. a nearly complete discoloration of the GC ink dispersion, whereas on the other extreme, the Ruby nanosecond laser is the least effective, leaving a significant residual absorption intensity. The samples treated with the Nd:YAG nanosecond laser show an intermediate effect, with the absorption features halfway in between those obtained with the Ruby Nano and the Nd:YAG picosecond lasers. The use of the array does not influence the spectra significantly: features obtained with or without array are quite comparable. A semiquantitative assessment of the efficacy of the lasers can be made by comparing the intensity of the most prominent absorption band located at 651 nm of the treated samples and the non-treated one. This corresponds to residual absorptivities of 22% after the Ruby nano treatment, 12% after the Nd:YAG nanoseconds treatments and 1% after the Nd:YAG picoseconds treatments. Laser treatments of phthalocyanines can be characterized also by a swap of intensity between the Q-band peaks at 651 nm and 727 nm^12^, which corresponds to a transition from the crystallographic alpha to beta phase of Cu-phthalocyanine and is promoted by heating thin solids^20^. No swap of intensity is observed in any of the present cases, hinting that the thermal associated events do not induce any changes in the π–π stacking arrangements of the present copper phthalocyanine moieties. As for the intensity increase in the 500–550 nm range, an analogous effect was observed when irradiating paper tinted with Heliogen Grün L8730, a paint based on PG7, with a polychromatic light generated by a Hg medium lamp 375 nm and generally attributed oxidative photobleaching processes and products^21^. Though differences between polychromatic and laser lights must be properly taken into account, it is possible that laser treatments of pigments and additives may generate species and/or species associations which absorb green visible light. It must also be considered that some of the additives play the role of rendering the pigments relatively stable in aqueous solution. The fragmentation of these additives and/or their separation from the pigments may increase the turbidity of the solutions, with consequent overall increase of the spectrum intensity and broadening of the spectral features.

### Pigment fragments and siloxanes are common to all laser treatments, hydrocarbons are laser treatment dependent

The GC-mass spectrometry of the treated samples reveals an overall complex scenario. The fragment compounds obtained in the 5 laser treatments are comparatively reported in Table 2 and can be divided in three main classes, pigments-related, hydrocarbons and siloxanes^13^. In addition, a colour code is adopted in Table 2, using blue for fragment compounds detected upon one of the laser treatments only, orange if the compounds appear in 2 to 4 treatments, dark green if they appear upon all laser treatments. Light green is used for siloxanes, which are also present upon each laser treatment. Due to the large number of hydrocarbons present, those with main chain length of 5 or more carbon atoms are reported in the Supplementary Information in Tables grouped by chain length.

**Table 2:**
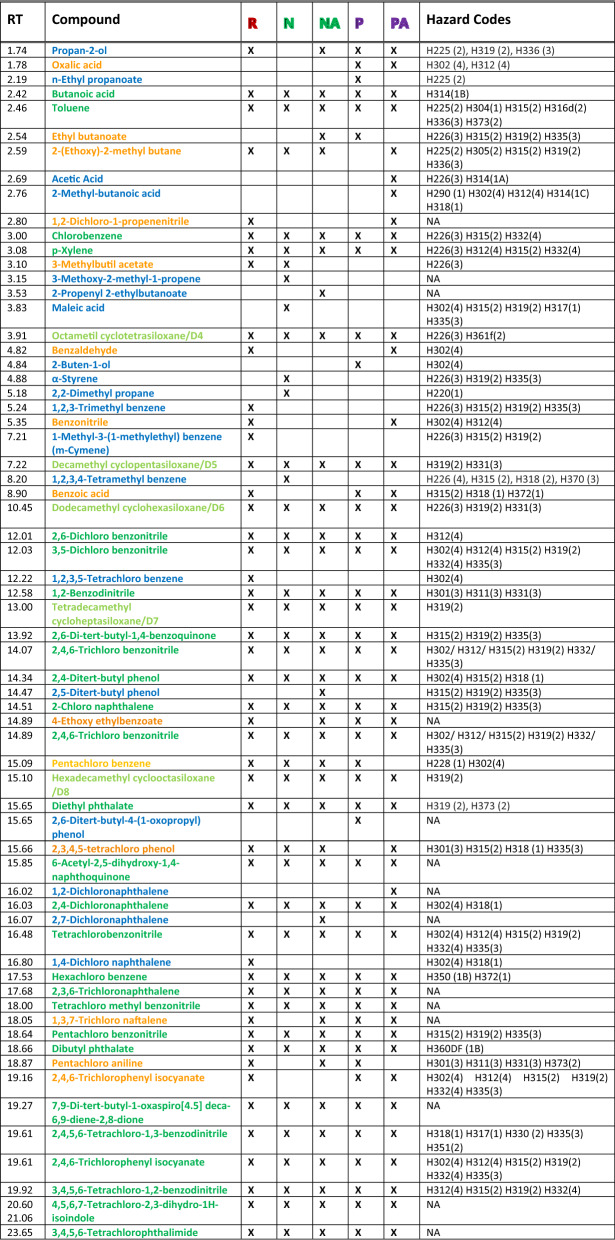
Fragment compounds produced upon different laser treatments of the GC ink:  = RubyNano,  = Nd:YAGNano,  = Nd:YAGNanoArray,  = Nd:YAGPico,  = Nd:YAGPicoArray.

The symbol **X** indicates the presence of the fragment compound. The fragment compounds reported in dark green are present upon each laser treatment, the orange ones in some of the treatments, the blue ones only in one. Siloxanes are reported in light green. The hazard codes are reported in the rightest column. NA implies no hazard code is available.

Several chlorinated compounds are generated upon laser treatments which can be related to the phthalocyanine fragmentation. The production of mono- di-, tri- and tetra-chlorinated benzene additionally carrying one or two –CN moieties is common to all treatments, as well as the formation of pentachlorobenzonitrile and hexachlorobenzene. Additional compounds are dichloronaphthalene (several isomers with the chlorine atoms in different positions), tetracholorophthalimide and 2,4,6-trichlorophenyl isocyanate. Health risks associated to these compounds include H312 (harmful in contact with the skin) and H315 (may cause skin irritation), as well as conditions of toxicity (H331), harmfulness (H302, H332) and irritation (H315, H319, H335). Two of them, however, present more serious drawbacks: hexachlorobenzene, which may cause cancer (H350) and damage to organs (H372) and 2,4,5,6-tetrachloro-1,3-benzodinitrile, which is suspected causing cancer (H351), it is fatal if inhaled (H330), and may cause allergic reactions (H317). It must be added that hexachlorobenzene did not emerge upon Nd:YAGNano laser treatment at lower fluence and longer irradiation time^14^. Benzene derivatives common to all laser treatments are diethylphthalate and dibutylphthalate. The origin of these fragment compounds can be twofold. They can be ascribed to the PG7 fragmentation, or be a residual of the initial phthalocyanine synthesis, which is carried out by condensation of phthalic anhydride with urea and CuCl_2_^22^. The latter hypothesis is supported by the absence of chlorine atoms on the phthalate, usually introduced in a following synthetic step. Their presence is of particular concern since their associated hazards are H373 (may cause damage to organs) and H360 (may damage fertility or the unborn child), respectively. Also chlorinated naphthalenes may be both the outcome of a rearrangement ensuing the laser treatment or a synthesis by-product. One of the isomers, the 2,4-dichloronaphthalene is common to all laser treatments, whereas 1,2-dichloronaphthalene emerges upon Nd:YAGPicoArray treatment, 2,7-dichloronaphthalene after Nd:YAGNanoArray and 1,4-dichloronaphthalene after RubyNano treatments only. Other chlorinated compounds of particular concern emerge exclusively upon some treatments. This is the case of pentachloroaniline which is particularly alarming because it may damage organs (H373).

Cyclic siloxanes D4, D5, D6, D7 and D8 are present upon each laser treatment, thus indicating that they have been added as ink additives and that they are resistant to all the laser treatments, when dispersed in water.

Of all siloxanes, D4 is the most harmful, since it is suspected of damaging the unborn child (H361).

Hydrocarbons and, to some extent, aromatic compounds are likely present as pigments additives, because of the pigments surface treatments, coating and encapsulation, which are usually performed during synthesis or post-synthesis with the purpose of enhancing dispersibility, decreasing powder cohesion^23^, inhibiting crystal growth^24^ and providing higher affinity with binders or liquids^25^. As far as the GC ink is concerned, neither the bottle label, nor the associated SDS report the presence of hydrocarbons. Nonetheless they were already previously detected in a green ink by the same brand upon low intensity treatment with Nd:YAG nano laser^14^. In the present case, butanoic acid, toluene, p-xylene, 2,6-di-tert-butyl-1,4-benzoquinone and 2,4-ditert-butyl phenol generate or emerge upon all treatments. The associated risks include skin and eye damage and/or irritation. Mostly toluene can be dangerous since it is classified with hazard statements H304, H373, i.e. lethal if swallowed or inhaled, and may cause damage to organs. As far as higher hydrocarbons are concerned, the partition with the laser treatments is more varied (Tables in the SI). There are at least 14 derivatives of the pentanoic acid (valeric acid), which include esters, alcohols, aldehydes, ketones, and alkenes, but only 2 of them can be found after all 5 treatments, the isopentyl acetate and the 2,2,4-trimethyl-3-carboxyisopropyl pentanoic acid isobutyl ester. All the other derivatives are products of one single treatment, with a larger frequency for the Nd:YAGNano irradiation. A complete comparative assessment of the toxicity is limited by the lack of information on some of the hydrocarbon derivatives. Similarly, 14 derivatives of hexanoic acid were observed, only 4 of which are common to all the treatments. The remaining 12 emerge as product of a single treatment or in 2 or 3 of them, again with a slight prevalence of products occurrence upon Nd:YAGNano treatment. All-in-all 61 different hydrocarbons emerge upon Nd:YAG:Nano treatment, 54 after Nd:YAG:NanoArray, 42 after Nd:YAG:Pico, 47 after Nd:YAG:PicoArray and 42 after RubyNano irradiation, respectively. The most harmful among them carry the codes H304 (may be lethal if swallowed or inhaled), H332 (harmful if inhaled) and H311 (toxic in contact with skin).

In general, the scenario of compound productions upon laser treatments is rather consistent, as far as pigment-related fragments and siloxanes are concerned, i.e. they are mostly present after all 5 treatments, and far more varied with respect to the hydrocarbons, whose presence is fairly treatment-dependent.

In this framework, we may attempt to distinguish compounds generated by the laser treatments or emerging after laser treatments. The pigment PG7 is broken into fragments and the current observations demonstrate that all laser treatments provide sufficient energy to overcome the onset of the fragmentation process, thus providing the same compounds in all cases. Hydrocarbons are embedded with the pigment together with other additives and the type of laser seems to be only one of the factors that concurs to make them evident. Siloxanes emerge upon all treatments, but their presence is favoured by the polar environment (water dispersion)^13^. It must be added that none of the species detected by GC-mass analysis absorbs in the 500–550 nm range of the visible spectrum. Therefore, the minor increase of intensity of the in visible spectrum is either related to a species not detectable by GC-mass or (more likely) it is due to solution turbidity.

### Fibers are produced upon picosecond lasers treatments, very large fibers are achieved in array mode

**Figure 2:**
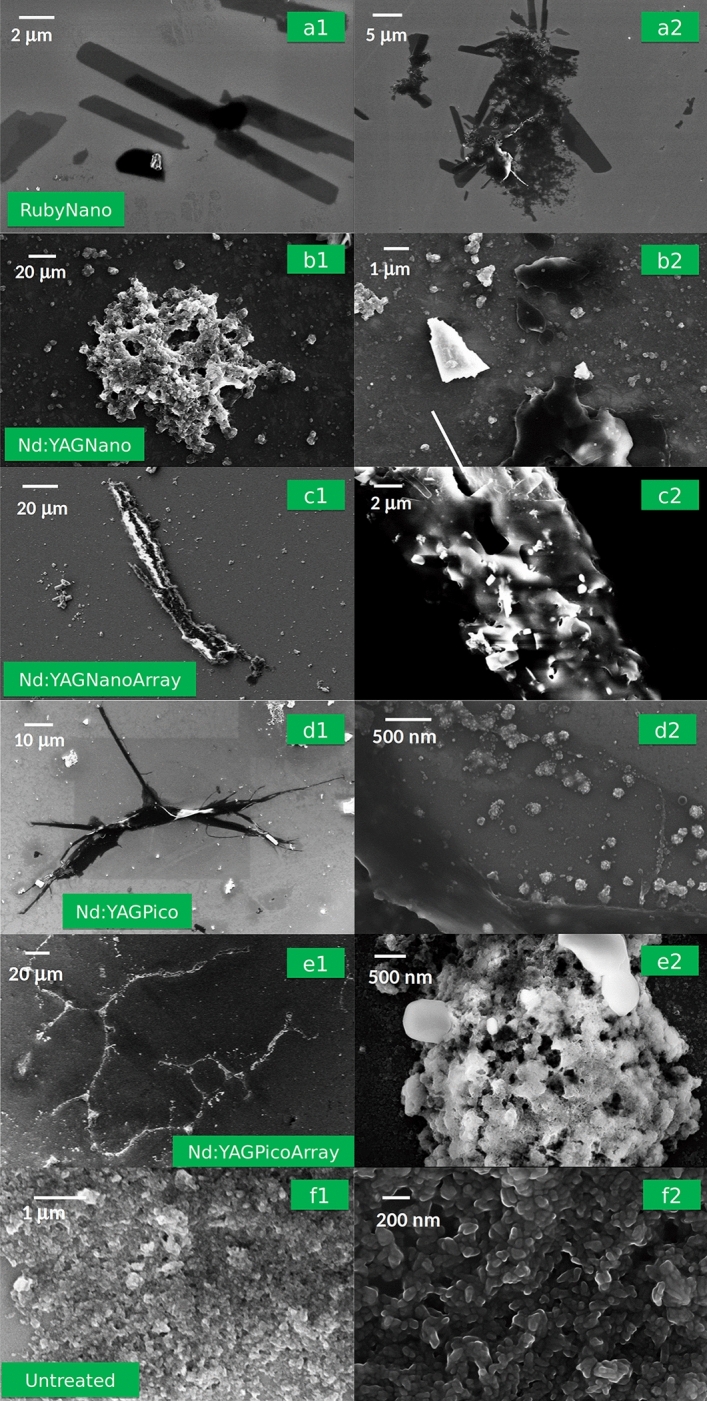
SEM images of GC ink treated with different lasers: (**a1,a2**) RubyNano, (**b1,b2**) Nd:YAGNano; (**c1,c2**) Nd:YAGNanoArray; (**d1,d2**) Nd:YAGPico; (**e1,e2**) Nd:YAGPicoArray, and untreated: (**f1,f2**).

The interaction of the laser with ink dispersion typically produces both fragmentation and re-aggregation of the ink particles^14^. This general trend is observed also in the current set-ups. Indeed, nanoparticles below 20 nm and down to 1–2 nm are detected for all treated samples. The aggregation features, however, are peculiar to the different treatments and an overview of the various morphologies is summarized in Fig. 2, including untreated GC ink sample for comparison purposes.

Pure phthalocyanines aggregate by π–π interactions between macrocycles which induce a stacking. The macroscopic appearance of the stacks, however, depends on the mutual positions of non-consecutive macrorings and can give rise to several shapes. When dealing with phthalocyanine based tattoo inks, several factors may concur to determine additional variability to the aggregation procedure. The phthalocyanines present in the investigated green ink are halogenated on the peripheral positions of the macrocycles, thus adding sterical hindrance which conditions the stacking pattern. Additives such as siloxanes and hydrocarbon are present, which survive the fragmentation process and can be conglomerated in the aggregates. Most importantly, the local temperature in the water dispersion largely fluctuates during the treatments because of the pulsed nature of the Q-switch lasers, which causes heating during the pulse followed by cooling during the pause. Furthermore, a temperature gradient occurs through the dispersion as a function of the spot size, beam structure and repetition rate. The irradiation with a nanosecond Ruby laser yields very distinctive thin and extended structures, like laminae. They have a preferred growth direction, resulting in an asymmetrical shape (Fig. 2a1) extending a few microns in the longest direction. Multiple laminae with different orientation may gather to provide larger structures such as in Fig. 2a2. Occasional blocks extending over tens of microns are also observed. The Nd:YAG nanosecond laser treatment is characterized by features related to beam piercing (Fig. 2b1) of the ink mass and the presence of blocks with dimensions in the order of 1–5 μm as well as layered structures (Fig. 2b2), similarly to what was found in previous investigations^14^ for the dried ink dispersed in water. Some differences in the layered structure, which is more extended in the present case, can be ascribed to operational differences, such as the fluence and total irradiation time. Very long linear fibers, up to 0.1 mm length and 15 μm diameter, are dominant shapes when the Nd:YAG laser is operated in NanoArray mode (Fig. 2c1).The magnification of the fibers reveals the presence of surface nanoparticles, thus suggesting an extensive particle conglomeration to form elongated structures. In addition, blocks with irregular shapes and heterogeneous sizes, up to 50 μm in the longest direction arise (Fig. SI3a of the Supplementary Information).

Elongated fiber-like structures are dominant shapes also for the Nd:YAG picosecond treatments (Fig. 2d1, e1), though with significant differences in the inner structures for the normal and the array modes (Fig. 2d2,e2). The Nd:YAGPico sample presents 100 micron long fibers, contoured by 30 micron long spikes (or quills). A large magnification of the fibers reveals the inherent presence of agglomerates of 200–300 nm diameter, made of particles with dimensions around 30 nm. The fiber structure achieved with the Nd:YAGPicoArray sample is far more extended and textured. The latter reaches 700–800 micron, with several branches and nodes at the connection points (Fig. 2e1). The magnification of one of the nodes in Fig. 2e2 indicates the presence of extensively melted structures, with both porous formations and compact grains. In addition, roundish shapes of 60–300 nm diameter are observed for both samples (Fig. SI3b of the Supplementary Information). For comparison purposes, the SEM images of the untreated GC ink sample are reported in Fig. 2f1,f2 at different magnifications, showing the characteristic extended sheath with incorporated roundish and elongated grains as already previously observed^14^. In general, the formation of fibers has likely a twofold origin. From one side phthalocyanines tend to aggregate in fibers due to π–π stacking, which eventually result in long superstructures, as observed by Kihara et al.^19^. On the other hand, siloxanes are bound to melt in vitreous fibers upon heating. Quite likely the elongation is due to interplay of the two phenomena at a different extent depending on the local ratio phthalocyanine/siloxanes, on temperature and temperature gradient as a function of the pulses.

In summary, picoseconds laser treatments of the green ink water dispersion tend to produce fibers (Nd:YAG) whereas after irradiation with the nanosecond Nd:YAG laser those morphologies are absent, or laminae are produced (Ruby). However, when lasers are operated in array mode, the nodes seem to act as connection points of agglomerates which cause further elongation. This implies long branched fibers for the Nd:YAGPicoArray and long linear ones for the Nd:YAGNanoArray. Additional residuals produced by Nd:YAG treatments are rather homogenous for the picosecond treatments (both normal and array mode) and heterogenous for nanosecond ones (both normal and array mode).

### The residual aggregates in water dispersions are laser treatment dependent

DLS measurements provide complementary information, with respect to SEM images. In particular, they give indications on the average size of the particles suspended in the water dispersions upon the various treatments. In addition, the polydispersity index (PDI) provides an indication of the samples heterogeneity. The DLS measurements are analysed by the cumulant method, which typically provides the overall average size and the PDI. Furthermore, DLS data analysis by the intensity-weighted method using NNLS algorithm gives an insight on the populations’ distributions. The DLS analyses are reported in Table 3 while the plots of the intensity weighted size distribution are presented in Fig. SI4 of the Supplementary Information. The DLS measurements of the RubyNano sample indicates a single population with an average size, 160 nm, only slightly larger than the non-treated sample and with a comparable PDI index. The average size values calculated by the NNLS method are slightly higher in both samples, the Ruby and the untreated sample. On the basis of the obtained results the ruby treatment yields the least absorbance reduction and the most peculiar shapes of solid residues. It may be hypothesized, that the suspended particles belong to residual non-treated sample, whereas treated ink deposits as lamellae and cannot be detected by DLS measurements. The samples treated with a nanosecond laser (Nd:YAGNano and Nd:YAGNanoArray) are the most heterogeneous ones, with a PDI of 0.535 and 0.572, respectively. Also the average size of the populations are the largest in these two samples, with components beyond the micron range and 10 micron range, respectively. More in detail, by NNLS size distribution analysis, the Nd:YAGNano sample is characterized by three populations (Fig. SI4), the most abundant one having an average size beyond the micron, along with populations of 244 nm and 70 nm. This corresponds to an average size centered at 270 nm according to the cumulant method.

**Table 3:** Average hydrodynamic diameter (2R_H_) from DLS measurements on the green ink Green Conc. in H_2_O, treated with different lasing setups, determined by cumulant analysis and intensity-weighted size distribution by NNLS algorithm.

Sample	2R_H_ (nm) [cumulant]	PDI	2R_H_ (nm) Peak 1—NNLS	2R_H_ (nm) Peak 2—NNLS	2R_H_ (nm) Peak 3—NNLS
RubyNano	160 ± 0.8	0.240 ± 0.003	195 ± 12		
Nd:YAGNano	270 ± 10	0.535 ± 0.110	60 ± 13	240 ± 30	1400 ± 90
Nd:YAGNanoArray	1460 ± 18	0.572 ± 0.072	843 ± 40		> 10 micron
Nd:YAGPico	311 ± 43	0.421 ± 0.045	62 ± 14	302 ± 43	
Nd:YAGPicoArray	276 ± 2	0.432 ± 0.008	58 ± 15	300 ± 11	
Non-treated	126 ± 2	0.265 ± 0.025	155 ± 3.5		

Data represents the average value and standard deviation over three repeated measurements.

The largest average size distribution and PDI is observed for the Nd:YAGNanoArray sample. In the latter NNLS analysis indicates a population centered at 843 nm, along with a second population exceeding 10 μm. This corresponds to an overall average size of 1460 nm obtained by the cumulant method and a PDI of 0.572. Both Nd:YAGPico and Nd:YAGPicoArray treatments yield more homogenous particle size distributions, with a PDI of 0.42/0.43. The size of the populations are also rather similar ranging about 300 nm. In addition, the NNLS method singles out a population characterized by smaller particles with dimensions in the order of 60 nm.

The DLS measurements reflect the SEM observations, especially as far as the agglomerates are concerned, taking into account that structures such as lamellae and long fibers are beyond the detection range of DLS. In general, both SEM and DLS indicate heterogeneous structures of sizes up to several microns in the case of nanosecond treatments (see for instance, Fig. SI3a) and rather homogenous structures for picosecond treatments (Fig. SI3b), with comparable sizes according to the two DLS data analysis methods (60–300 nm). A possible shape-determining mechanism may be described by an initial formation of agglomerates, which possibly merge further to form fibers. Size and homogeneity of the agglomerates are mostly influenced by the initial pulse duration and associated peak temperature. The subsequent growth into fibers is conditioned both by the type of initial agglomerates, and the operational mode, i.e., whether it is normal or array. The wavelength of the laser might also play a role, with the RubyNano forming small, homogenous initial aggregates and lamellae. In general, the laser irradiation mainly triggers two mechanisms, eventually leading to the ink removal: i.e. photothermal and photoacoustic effects, both initiated by radiation absorption. The chromophore absorptivities at the Ruby (694 nm) and Nd:YAG (532 nm) wavelengths are different, the former being larger than the latter, with a consequent larger initial conversion ratio of light into thermal energy^12^. The subsequent development of the mechanical expansion waves is determined by the pulse energy, duration and frequency, which make Nd:YAG lasers, overall more efficient. The photochemical mechanism is only a minority channel, since both laser wavelengths do not correspond to resonant absorptions of the chromophore.

## Conclusions

The Nd:YAG picosecond lasers in normal and in array modes are the most effective in discoloring the green tattoo ink dispersions, displaying a 1% residual of the initial absorbance at the end of the treatment. They are also the setups providing the longest and most textured fibers, contoured by quills (normal mode), and a rather homogeneous size distribution of the residual agglomerates suspended in the dispersion. Nd:YAG nanosecond treatments provide a moderate discoloration (12% residual absorbance), a substantially different morphology, when operated in normal mode, with the generation of blocks and texture piercing, and in array mode, with the production of long and thick fibers. The size distribution of the agglomerates in dispersion is quite heterogeneous. The Ruby nanosecond treatment is the least effective in terms of dispersion discoloration (22% residual absorbance), it generates characteristic laminae-like structures, and the residual agglomerates in dispersion are only slightly larger than the untreated sample and with a homogenous size distribution, hinting a conspicuous content of untreated ink. The GC-mass spectra indicate that all treatments leave siloxanes, including the most harmful ones such as the D4, suspected of damaging the unborn child (H361). The pigment fragment products are similar upon all treatments, including toxic ones such as hexachlorobenzene and 2,4,5,6-tetrachloro-1,3-benzodinitrile. Organs damaging contaminants such as pentachloro aniline, instead, emerge only in some of the laser treatments. Finally, the presence of hydrocarbons is treatment dependent, with a larger production of fragments of different types, using the Nd:YAGNano laser. In addition, some distinction of classes of products can be made, with linear alcohol molecules, for instance, mostly detected upon Nd:YAGNano treatment, branched alcohols upon Nd:YAGNanoArray.

## Materials and methods

### Ink samples

The ink Green Concentrate by Eternal Ink, Inc. was purchased in a regular shop for tattoo supply in Rome (Italy). All chemicals used in this investigation were of reagent grade and used without any further purification. Ethyl acetate was received from Merck. All dispersions were prepared by dilution with deionized water followed by 30 min sonication at 40 kHz, at a concentration of 0.09 mg/ml, which corresponds to a homogeneous dispersion, with an eye-appreciable colour intensity and an 18 h room temperature stability, before sedimentation arises.

### Laser equipment and treatments

The laser treatments were performed either with a ruby laser (694 nm) or with a Nd:YAG laser (532 nm) by DEKA Laser (Discovery Pico Plus). The latter can be operated both in the nanosecond (6 ns) or picosecond (370 ps) range, in single beam or in array mode, with 180 nodes. The total irradiation is the same for all samples, i.e. 2 kJ. The summary of the operational conditions is reported in Table 1.

### Discoloration analysis

The laser treatment was initially carried out with the Nd:YAG laser operated in the picosecond range, until the dispersion was visually discoloured. The corresponding value of total irradiation energy was subsequently applied to all the other treatments. The resulting dispersions were consequently analysed by UV–Vis spectrometry, using a Perkin Elmer Lambda 950 spectrophotometer, in the 250–800 nm range, using quartz sample holder with an optical path length of 1 mm.

### Fragmentation analysis

The treated inks were extracted with ethyl acetate. The analyses were, then, carried out by gas chromatography-mass spectrometry, employing a Trace GC Ultra gas chromatograph (Thermo Scientific, Waltham, MA, USA) equipped with a TriPlus autosampler unit and coupled to a TSQ Quantum Triple Quadrupole GC–MS/MS spectrometer (Thermo Scientific, Waltham, MA, USA). An XLB-ms fused silica capillary column (Varian, Inc.), 60 m × 0.25 mm, i.d. 0.25 μm film thickness was used for chromatographic separation, with hydrogen as carrier gas at 3 mLmin^−1^ flow rate. 1 μl solution was injected in splitless mode at 250 °C. The oven program consisted of an isotherm at 90 °C for 5 min, a temperature ramp of 10 °C min^−1^ up to 280 °C, which was hold for 5 min. The MS was operated in positive electron ionization (EI+) mode, with electron energy of 70 eV and an emission current of 50 μA. The acquisition was in scan mode in the range 35–600 m/z in 0.2 s. The transfer line and ion source temperatures were kept at 290 °C and 300 °C, respectively.

### Morphology analysis by SEM

A drop of the dispersion upon each laser treatment, as well as of untreated sample, was deposited on a silicon-wafer sample holder for scanning electron microscopy (SEM) analysis. SEMs images were collected with a Zeiss Auriga Field Emission-Scanning Electron Microscope instrument operating at 7 kV. The EDX analyses were made by coupling the Field Emission Scanning Electron Microscope (SUPRA™ 35, Carl Zeiss SMT, Oberkochen, Germany).

### Average size analysis of the ink dispersions by DLS

Determination of hydrodynamic size and size distribution of ink samples was carried out by Dynamic Light Scattering (DLS) by a Malvern NanoZetaSizer apparatus (Malvern Instruments LTD, UK) equipped with a 5 mW HeNe laser, temperature control by a Peltier system and backscattering detection. This configuration is less sensitive to multiple scattering effects and dust than the 90° geometry. Measurements were performed at 298 ± 0.5 K and repeated three times each. The distribution of apparent hydrodynamic radii R_H_ is obtained by analysis of the DLS autocorrelation function of scattered intensity. As previously reported^13,14^ the cumulant method has been considered to get the average hydrodynamic size R_H_ and the polydispersity index (PDI)^26^. This method yields the most direct and robust determination of the average values of the hydrodynamic size, because it relies on the initial part of the autocorrelation function where the signal-to-noise ratio is largest and it does not presuppose a certain size distribution of the sample. Unfortunately, the so-determined average R_H_ may not necessarily be a significant representation when the PDI values are larger than 0.2–0.3, which indicates a broad size distribution where more than one maximum could be present. In this case, additional detailed size distribution may be provided by intensity-weighted NNLS algorithm^27^. Note that in the intensity-weighted analysis, the obtained R_H_ is biased on larger size because the scattered intensity is proportional to the sixth power of the particle size.

## Supplementary Information

Supplementary Information.
