# Supplementary material for: Comparative treatments of a green tattoo ink with Ruby, Nd:YAG nano- and picosecond lasers in normal and array mode
Source: Sci Rep. 2022 Mar 4;12:3571. doi: 10.1038/s41598-022-07021-w (PMC8897463; doi:10.1038/s41598-022-07021-w)
Supplement: Supplementary file 1 — Supplementary Information. [file 41598_2022_7021_MOESM1_ESM.docx]

# **Supplementary information**

**Comparative treatments of a green tattoo ink with Ruby, Nd:YAG nano- and picosecond lasers in normal and array mode**

Daniele Cecchetti^1#^, Elvira Bauer^2#,^ Ettore Guerriero^3^, Simona Sennato^4^, Pietro Tagliatesta^1^, Marco Tagliaferri^5^, Luca Cerri^6^, Marilena Carbone^1^*

^1^ Department of Chemical Science and Technologies, University of Rome Tor Vergata, Via della

Ricerca Scientifica, 1 – 00133 Rome, Italy

^2^ Institute of Structure of Matter, Italian National Research Council (CNR-ISM), Via Salaria km 29.3, 00015 Monterotondo, RM, Italy

^3^ Institute of Atmospheric Pollution Research, Italian National Research Council (CNR-IIA), Via Salaria km 29.3, 00015 Monterotondo, RM, Italy

^4^ Institute of Complex Systems, Italian National Research Council (CNR‑ISC), Sapienza Unit, and Physics Department, Sapienza University, P.le A. Moro 5, 00185 Rome, Italy

^5^ El.En. S.p.A., Via Baldanzese 17, 50041 Calenzano (Fi), Italy

^6^ Quanta System S.p.A., Via Acquedotto 109, 21017 Samarate (VA), Italy

*corresponding author: Marilena Carbone (carbone@uniroma2.it)

^#^ These two authors equally contributed to the paper

**Figure SI1** Photo of the laser Nd:YAG pico array taken on laser alignment burn paper Z3T ZAP-IT.

**Figure SI2** Photos of the GC ink dispersions before and after the various laser treatment. The label underneath each vial identifies the corresponding type of laser treatment.

The following tables outline the hydrocarbons produced upon different laser treatments of the GC ink, whose main chain contains 5 of more carbon atoms. In each table the fragment hydrocarbons are grouped by the length of the main chain, which is indicated on the heading. **R** indicates the RubyPico sample, **P** the Nd:YAGPico sample, **PA** the Nd:YAGPicoArray sample, **N** the Nd:YAGNano sample and **NA** the Nd:YAGNanoArray sample. The symbol **X** indicates the presence of the fragment compound upon laser treatment in the column where it appears. The fragment compounds reported in dark green are present upon each laser treatment, the orange ones are produced in some of the treatments, whereas the blue ones are present only upon one of the treatments. The hazard codes corresponding to each compound are reported in the rightest column. NA implies no hazard code is available.

**5C**

| **RT** | **Compound** | **R** | **N** | **NA** | **P** | **PA** | **Hazard Codes** |
| --- | --- | --- | --- | --- | --- | --- | --- |
| 2.05 | **3-Methyl-4-oxo pentanoic acid** |  | **X** |  |  |  | H319(2) |
| 2.69 | **Pentyl acetate** |  |  |  |  | **X** | H226(3de) |
| 2.73 | **4,4-Dimethyl-2-pentenal** |  | **X** |  |  |  | / |
| 3.10 | **Isopentyl acetate** | **X** | **X** | **X** | **X** | **X** | H226(3) |
| 3.43 | **3,4-Dimethyl-3-penten-2-one** |  |  |  | **X** |  | NA |
| 3.62 | **2,3,4,5-Tetramethyl-2-cyclopenten-1-ol** |  | **X** |  |  |  | NA |
| 4.42 | **Valeric acid** |  |  | **X** |  |  | H314(1B) |
| 5.35 | **Cyclopentane carbaldehyde** |  | **X** |  |  |  | H315(2) H319(2) H335(3) |
| 5.63 | **2,4,4-Trimethyl-1-pentene** |  |  |  |  | **X** | H225(2) |
| 5.64 | **2-Ethyl-4-methyl-1-pentanol** |  |  | **X** |  |  | H315(2) H319(2) H332(4) H335(3) |
| 5.97 | **2-(1-methylpropyl)-cyclopentanone** |  |  |  | **X** |  | NA |
| 11.18 | **1,2-Dimethyl cyclopentane** | **X** |  |  |  |  | H225(2) H315(2) H319(2A) H335(3) |
| 15.35 | **2,2,4-Trimethyl-3-carboxyisopropyl pentanoic acid isobutyl ester** | **X** | **X** | **X** | **X** | **X** | NA |

**6C**

| **RT** | **Compound** | **R** | **N** | **NA** | **P** | **PA** | **Hazard Codes** |
| --- | --- | --- | --- | --- | --- | --- | --- |
| 2.73 | **2,2-Dimethyl-2-hexene** |  |  |  |  | **X** | NA |
| 2.98 | **3-Methyl-3-hexene** |  |  |  | **X** |  | NA |
| 3.01 | **1,2,6-Hexanetriol** |  |  | **X** |  |  | NA |
| 3.33 | **4,4-Dimethyl-2-cyclohexen-1-ol** |  |  | **X** |  |  | NA |
| 3.48 | **2-Ethyl-1,3-hexanediol** |  |  | **X** |  |  | H318(1) |
| 3.93 | **1,3-Dimethyl cyclohexane** | **X** | **X** | **X** | **X** | **X** | H225(2) H315(2) H319(2) H335(3) |
| 4.42 | **Palmitic acid** |  |  |  |  | **X** | H331(3) H314(1C) H318(1) |
| 4.43 | **Hexanoic acid** | **X** | **X** | **X** | **X** | **X** | H331(3) H314(1C) H318(1) |
| 4.68 | **1,2,3,4-Tetramethyl cyclohexane** |  | **X** |  |  |  | NA |
| 4.82 | **3-Hexene-2,5-dione** | **X** | **X** |  |  | **X** | H302(4) |
| 5.65 | **3-Methyl cyclohexanone** |  | **X** |  | **X** |  | H226(3) H315(2) H319(2) H335(3) |
| 6.53 | **3,3,5-Trimethyl cyclohexan-1-one** | **X** | **X** | **X** | **X** | **X** | H319(2) H335(3) H315(2) H332(4) |
| 7.79 | **4,5-Dimethyl-4-hexen-3-one** | **X** | **X** | **X** | **X** | **X** | NA |
| 10.06 | **1-Ethyl-2,4-dimethyl-cyclohexane** |  | **X** |  |  |  | NA |

**7C**

| **RT** | **Compound** | **R** | **N** | **NA** | **P** | **PA** | **Hazard Codes** |
| --- | --- | --- | --- | --- | --- | --- | --- |
| 2.69 | **3,5-Dimethyl-1,6-heptadien-1-ol** |  | **X** |  |  |  | NA |
| 3.15 | **2-Nitro-2-hepten-1-ol** |  |  | **X** |  |  | NA |
| 3.35 | **4-Methyl-3-heptanone** |  | **X** |  |  |  | H226(3) H315(2) H319(2A) H332(4) H335(3) |
| 3.48 | **5-Methyl-3-heptanone** |  |  |  |  | **X** | H226(3) H319(2) H335(3) |
| 7.78 | **4-Methyl-4-hepten-3-one** | **X** | **X** | **X** | **X** | **X** | NA |
| 13.71 | **2,6,10,15-Tetramethyl heptadecane** | **X** | **X** | **X** | **X** | **X** | NA |

**8C**

| **RT** | **Compound** | **R** | **N** | **NA** | **P** | **PA** | **Hazard Codes** |
| --- | --- | --- | --- | --- | --- | --- | --- |
| 2.69 | **1-Octyn-3-ol** |  |  |  |  | **X** | NA |
| 3.43 | **2,7-Dimethyl octane** |  | **X** | **X** |  |  | NA |
| 5.16 | **6-Methyl octane** |  |  | **X** |  |  | NA |
| 6.14 | **5-Ethyl-2-methyl octane** | **X** |  |  |  |  | NA |
| 7.03 | **3,5-Dimethyl octane** |  | **X** |  |  |  | H226(3) |
| 10.95 | **2-Buthyl-1-octanol** |  |  | **X** |  |  | NA |

**9C**

| **RT** | **Compound** | **R** | **N** | **NA** | **P** | **PA** | **Hazard Codes** |
| --- | --- | --- | --- | --- | --- | --- | --- |
| 3.26 | **Nonane** |  |  |  | **X** |  | H226(3) H304(1) H315(2) H336(3) |
| 3.35 | **4-Methyl-3-heptanone** |  | **X** |  |  |  | H226(3) H315(2) H319(2A) H332(4) H335(3) |
| 3.48 | **5-Methyl-3-heptanone** |  |  |  |  | **X** | H226(3) H319(2) H335(3) |
| 3.53 | **4-Oxononanal** | **X** | **X** |  |  |  | NA |
| 3.60 | **Nona-3,5-dien-2-ol** | **X** |  |  |  |  | NA |
| 3.62 | **2-Nonenal** |  |  | **X** |  |  | H315(2) H319(2) |
| 5.35 | **4,4-Dimethyl-5-nonenal** |  |  |  |  | **X** | NA |
| 5.35 | **Nonyl 2,2-dimethylpropanoate** |  | **X** |  |  |  | NA |
| 7.66 | **2-Nonen-1-ol** |  |  |  | **X** | **X** | NA |
| 10.68 | **Nonanoic acid** | **X** | **X** | **X** | **X** | **X** | H315(2) H319(2) |

**11C**

| **RT** | **Compound** | **R** | **N** | **NA** | **P** | **PA** | **Hazard Codes** |
| --- | --- | --- | --- | --- | --- | --- | --- |
| 3.25 | **3,7-Dimethyl undecane** | **X** | **X** |  |  |  | NA |
| 3.27 | **2-Undecanol** |  |  | **X** |  |  | H315(2) H319(2) H335(3) |
| 5.99 | **Undecane** |  | **X** | **X** |  |  | NA |
| 7.02 | **3,5-Dimethyl undecane** | **X** |  |  |  |  | NA |
| 7.16 | **4,7-Dimethyl undecane** |  | **X** |  |  |  | NA |
| 7.65 | **2-Undecen-1-ol** |  | **X** |  |  |  | NA |
| 9.04 | **1-Undecanol** |  |  |  | **X** |  | H319(2) H315(2) |
| 9.14 | **Undecane** |  |  | **X** |  |  | NA |
| 11.26 | **4,6-Dimethyl undecane** |  |  |  | **X** |  | NA |
| 14.47 | **4-Ethyl undecane** |  |  | **X** |  |  | NA |

**12C**

| **RT** | **Compound** | **R** | **N** | **NA** | **P** | **PA** | **Hazard Codes** |
| --- | --- | --- | --- | --- | --- | --- | --- |
| 9.04 | **1-Dodecene** | **X** | **X** | **X** | **X** | **X** | H304(1) H315(2) |
| 9.15 | **Dodecane** |  | **X** |  |  |  | H226(3) H304(1) H319(2) H335(2/3) |
| 9.69 | **4-Dodecenol** |  | **X** |  |  |  | NA |
| 14.33 | **2,6,11-Trimethyl dodecane** | **X** | **X** | **X** | **X** | **X** | NA |
| 16.90 | **2,6,10-Trimethyl dodecane** |  | **X** | **X** |  | **X** | H304(1) |

**13C**

| **RT** | **Compound** | **R** | **N** | **NA** | **P** | **PA** | **Hazard Codes** |
| --- | --- | --- | --- | --- | --- | --- | --- |
| 15.10 | **1-Tridecanol** |  | **X** |  |  |  | NA |

**14C**

| **RT** | **Compound** | **R** | **N** | **NA** | **P** | **PA** | **Hazard Codes** |
| --- | --- | --- | --- | --- | --- | --- | --- |
| 12.53 | **Tetradecane** | **X** | **X** | **X** | **X** | **X** | H304(1) H315(2) H336(3) |
| 14.60 | **2,6,10-Trimethyl tetradecane** |  | **X** | **X** |  | **X** | NA |

**16C**

| **RT** | **Compound** | **R** | **N** | **NA** | **P** | **PA** | **Hazard Codes** |
| --- | --- | --- | --- | --- | --- | --- | --- |
| 2.86 | **2-Hexadecanol** |  |  | **X** |  |  | NA |
| 4.42 | **Palmitic acid** |  |  |  |  | **X** | H311(3) H314(1C) H318(1) |
| 11.25 | **Hexadecane** |  |  | **X** |  |  | NA |
| 15.20 | **1-Hexadecanol** | **X** | **X** | **X** | **X** | **X** | NA |

**17C**

| **RT** | **Compound** | **R** | **N** | **NA** | **P** | **PA** | **Hazard Codes** |
| --- | --- | --- | --- | --- | --- | --- | --- |
| 2.69 | **3,5-Dimethyl-1,6-heptadien-1-ol** |  | **X** |  |  |  | NA |
| 13.71 | **2,6,10,15-Tetramethyl heptadecane** | **X** | **X** | **X** | **X** | **X** | NA |
| 14.60 | **Heptadecane** | **X** |  |  |  |  | H304(1) |
| 17.56 | **1-Heptadecanol** |  | **X** |  |  |  | NA |

**18C**

| **RT** | **Compound** | **R** | **N** | **NA** | **P** | **PA** | **Hazard Codes** |
| --- | --- | --- | --- | --- | --- | --- | --- |
| 19.16 | **2-Methyl octadecane** |  |  | **X** |  |  | NA |

**19C**

| **RT** | **Compound** | **R** | **N** | **NA** | **P** | **PA** | **Hazard Codes** |
| --- | --- | --- | --- | --- | --- | --- | --- |
| 13.71 | **Nonadecane** | **X** | **X** | **X** | **X** | **X** | H304(1) |
| 16.38 | **Nonadecene** |  | **X** |  |  |  | NA |
| 16.89 | **2-Methyl nonadecane** |  | **X** |  |  |  | NA |
| 20.80 | **10-Methyl nonadecane** | **X** | **X** | **X** | **X** |  | NA |

**22C and more**

| **RT** | **Compound** | **R** | **N** | **NA** | **P** | **PA** | **Hazard Codes** |
| --- | --- | --- | --- | --- | --- | --- | --- |
| 2.97 | **Erucic acid** |  |  | **X** |  |  | NA |
| 17.57 | **10-Eicosene** |  |  | **X** | **X** | **X** | NA |
| 16.89 | **2-Methyl eicosane** |  |  |  |  | **X** | NA |
| 21.20 | **Heptacosane** | **X** | **X** | **X** | **X** | **X** | NA |
| 21.63 | **1-Heptatriacontanol** | **X** | **X** | **X** | **X** | **X** | NA |
| 21.80 | **Docosanol** |  |  |  | **X** |  | NA |
| 21.80 | **13-Eicosenoic acid** |  | **X** |  |  |  | NA |
| 21.85 | **1-Eicosanol** | **X** |  | **X** |  | **X** | NA |

**Figure SI3**. SEM images of GC ink treated with a) Nd:YAGNanoArray, b) and Nd:YAGPicoArray.


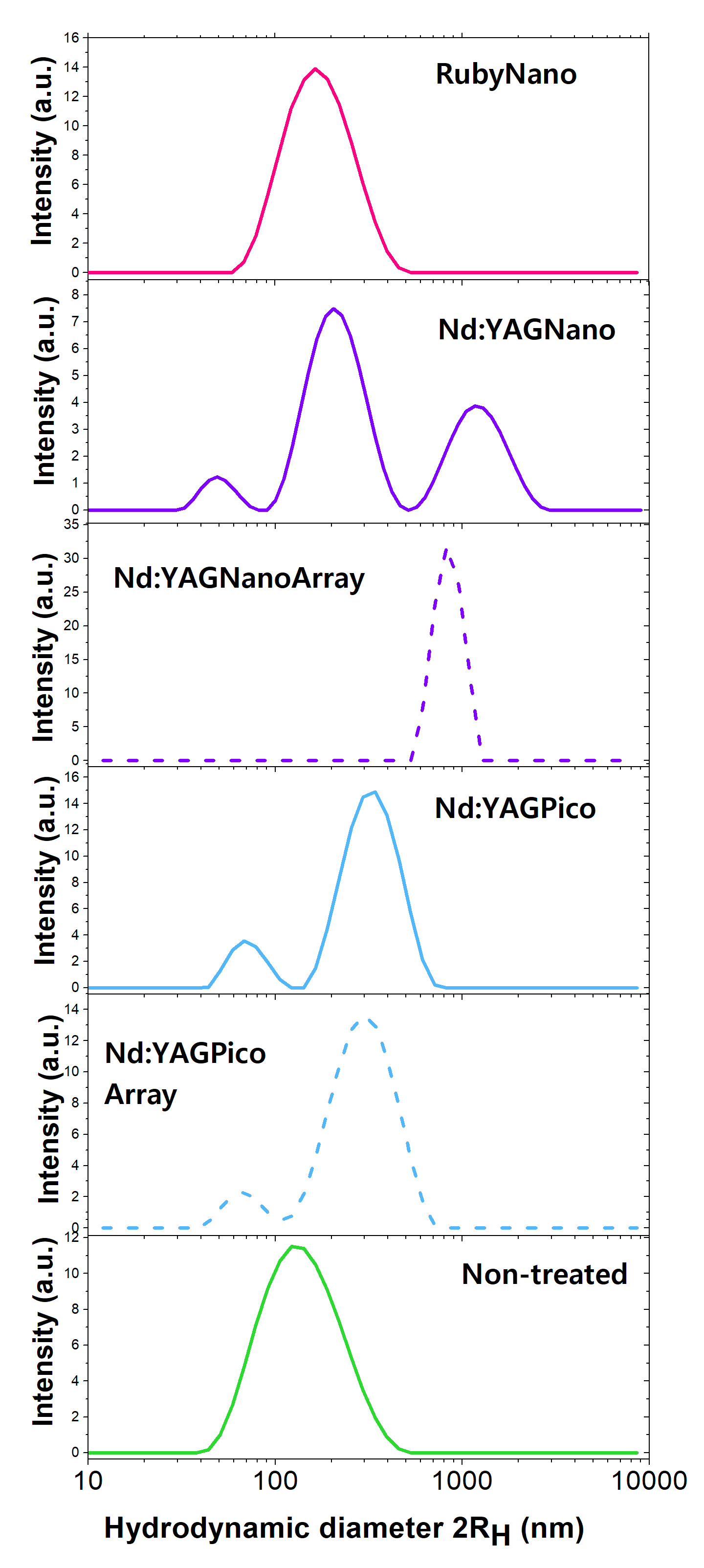


**Figure SI4** Intensity weighted size distribution obtained by NNLS analysis of DLS measurements for the different ink dispersion upon laser treatments: red solid line = nanosecond ruby laser, violet solid line = nanosecond Nd:YAG, violet dashed line = nanosecond Nd:YAG with array, light blue solid line = picosecond Nd:YAG, light blue dashed line = picosecond Nd:YAG with array. In the bottom panel, the size distribution of the non-treated sample is reported as a reference (green solid line).
